# Factors Affecting the Implementation and Acceptance of the Cocoon Strategy in the NICU in a Tertiary Center in Türkiye

**DOI:** 10.3390/vaccines12030319

**Published:** 2024-03-18

**Authors:** Şeyma Karatekin, Selda Hançerli Törün, Ebru Şenol, Salih Çağrı Çakır, Gülbin Gökçay

**Affiliations:** 1 Department of Child Health and Disease, Faculty of Medicine, Samsun University, Samsun 55080, Türkiye; 2 Social Pediatrics Doctorate Programme, Institute of Child Health Department, İstanbul University, Istanbul 34104, Türkiye; drebrusenol@gmail.com; 3 Division of Pediatric Infectious Diseases, Department of Pediatrics, İstanbul Faculty of Medicine, İstanbul University, Istanbul 34104, Türkiye; selda.hancerli@istanbul.edu.tr; 4 Neonatology, Faculty of Medicine, Bursa Uludağ University, Bursa 16059, Türkiye; salihcagri@gmail.com; 5 Department of Social Pediatrics, Institute of Child Health, İstanbul University, Istanbul 34104, Türkiye; drgulbin@gmail.com

**Keywords:** vaccination, pertussis, cocoon strategy, newborn, neonatal intensive care units

## Abstract

Pertussis is an important cause of mortality and morbidity in infancy. It is recommended that close contacts of the baby be vaccinated with Tdap, and this practice is called the cocoon strategy. This study aimed to investigate the applicability of the cocoon strategy and to determine the factors affecting the process. Mothers of babies who were hospitalized in the neonatal intensive care unit were included in the study. In the first stage, a face-to-face questionnaire was given to the mothers to measure their level of knowledge about whooping cough and its vaccine. In the second stage, written and verbal information about the cocoon strategy was given, and then vaccination intentions for Tdap were learned. In the third stage, all mothers were contacted 3 weeks after and asked whether they had received a Tdap vaccination and why. Of these mothers, 68% could not answer any questions about pertussis disease and vaccines correctly. After the information, 35% (n = 78) of the mothers stated that they were considering getting vaccinated, while only 2% (n = 5) of the mothers were able to get the Tdap vaccine. The most important reasons for not getting vaccinated were a lack of time (24%) and the cost of vaccination (23%). It is predicted that Tdap vaccination rates may increase if the cost of vaccine, availability of vaccine, and the access of mothers to the vaccine application are facilitated.

## 1. Introduction

Pertussis, a respiratory tract infection caused by Bordetella pertussis, is transmitted through respiratory droplets. Globally, it continues to be a significant cause of mortality and morbidity in infancy [1]. Pertussis is highly contagious among household contacts, infecting up to 90% of unvaccinated individuals at home, and untreated individuals can remain infectious for up to 3 weeks [1]. The disease tends to be more severe especially in infants under 6 months old, particularly in preterm babies, and in those who have not yet been vaccinated [2]. Approximately 75% of deaths occur in infants under 3 months old [3].

According to World Health Organization (WHO) estimates in 2013, there were 63,000 deaths annually in children under 5 years of age due to pertussis [1]. In the United States, 48,277 cases of pertussis and 20 associated deaths were reported in 2012 and it was observed that the number of cases was the highest in recent years. The cases primarily peaked during infancy and secondarily during adolescence [3]. In Türkiye, despite a significant decrease in the burden of the disease in recent years, deaths attributed to pertussis continue to be reported in the infancy period [4,5]. Although notification problems in developing countries are taken into consideration, a total of 10 deaths due to pertussis were reported in our country between 2012 and 2017 [6].

In our country, acellular vaccines were licensed in 2008. Currently, within the framework of the Expanded Program on Immunization (EPI), a total of five doses of acellular pertussis vaccine are administered, including primary immunization at 2 months, 4 months, and 6 months, and booster doses at 18 and 48 months [6]. While the coverage of three doses of acellular pertussis vaccine is 86% globally, it has been reported as 96% in our country [6].

The cocooning strategy, defined as vaccinating close contacts of the newborn with Tdap, is recommended by the American Academy of Pediatrics and Centers for Disease Control and Prevention (CDC) to reduce pertussis cases during infancy [2]. The Advisory Committee on Immunization Practices (ACIP) has been recommending the vaccination of close contacts (parents, grandparents, healthcare workers, babysitters, etc.) of infants under 1 year of age with Tdap since 2006 [7]. While current vaccination recommendations from the committee emphasize pregnancy vaccines for pertussis prevention, the suggestion of vaccinating close contacts, known as the cocooning strategy, continues [8]. However, challenges may arise in the implementation of the cocooning strategy. It has been observed that Tdap vaccination rates vary depending on factors such as the location and timing of vaccination, vaccine cost and accessibility, healthcare workers’ awareness, and parental education about the topic [9,10,11].

This study encompasses mothers of newborns who are admitted in the neonatal intensive care unit. The aim is to investigate the feasibility of the cocooning strategy, which is not yet widespread in our country, and to identify the factors affecting the implementation process.

## 2. Materials and Methods

### 2.1. Study Design and Setting

This study is designed as a cross-sectional and descriptive study. Mothers of newborns who were treated in the neonatal intensive care unit (NICU) of Samsun Women’s and Children’s Diseases Hospital between 5 April 2021 and 18 November 2022 were included in this study. This hospital is a tertiary public hospital with approximately 4000 deliveries annually. The NICU of the hospital has a capacity for 45 patients, who are categorized as level 1, 2, or 3. Among the private, university, and public hospitals in Samsun, this NICU has the highest patient capacity. The study included mothers of newborns treated at any level of care.

**Figure 1:**
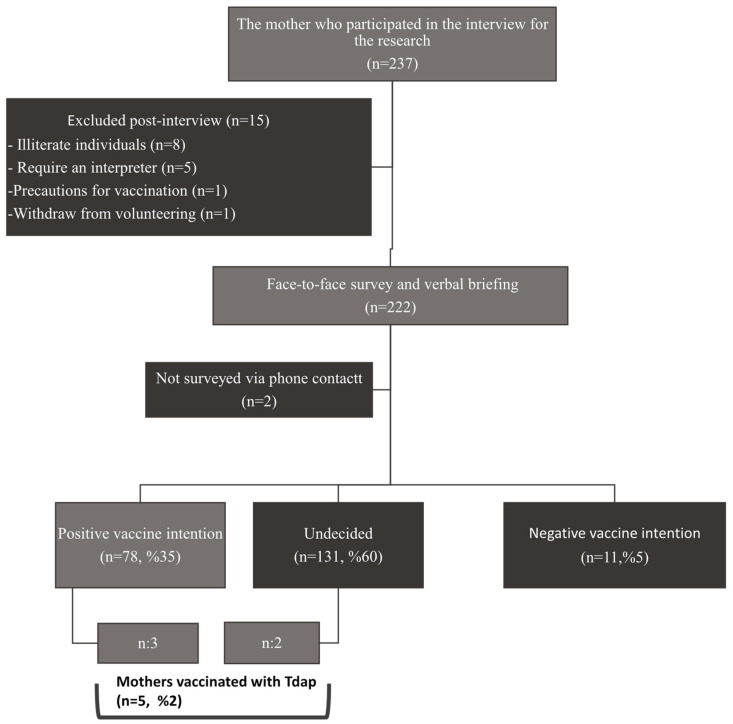
Flowchart of pertussis study.

Exclusion criteria for this research include mothers who are illiterate, require an interpreter, have a contraindication/precaution for vaccination, have received pertussis vaccine during pregnancy, are mothers of newborns abandoned to social services, mothers of newborns admitted to the NICU with COVID-19, mothers of newborns with multiple congenital anomalies incompatible with life (recommended for medical termination), and mothers of deceased newborns. The flowchart of this study is presented in Figure 1.

### 2.2. Data Collection

This research was conducted in the family information room within the NICU on designated information days (Monday–Wednesday–Friday) and times for discussing the health status of their babies. The information sessions were conducted by a single pediatrician. This survey was conducted according to the guidelines of the Declaration of Helsinki after prior approval by the Clinical Research Ethics Committee of Health Sciences University Samsun Training and Research Hospital, with decision number 2021/5/14. The research was planned in three stages.

Stages 1: Within 1 week of the newborns’ admission to the NICU, meetings were arranged with their mothers. Mothers (n = 237) were provided detailed information about this research. Volunteering mothers (n = 236) were asked four questions. These four questions queried about any serious allergic reactions (anaphylaxis) to vaccines, medications or substances, coma, confusion, or prolonged seizures occurring within 7 days after the previous Diphtheria Tetanus Acellular Pertussis (DTaP) vaccine, neurological diseases such as Guillain-Barre syndrome developing within 6 weeks after previous vaccinations, and diagnosis of any previous diseases such as epilepsy, etc. Mothers without contraindications/precautions for vaccination (n = 222) provided written consent through informed consent forms. The first part of this survey, consisting of 31 questions and lasting approximately 10 min, was administered face-to-face by the researcher physician.

Stages 2: After completing the survey, mothers (n = 222) received a five-minute verbal briefing on pertussis disease and cocooning strategy. Clinical symptoms of pertussis, transmission routes, and methods of protection against pertussis were explained. After explaining the purpose of the cocooning strategy, mothers and close contacts of the baby were advised to get vaccinated with Tdap. Following the verbal briefing, the family information form, containing the same information, was given to the mother and she was asked to read it. Any additional questions were addressed. At this point, mothers were asked about their vaccine intention. All mothers were provided with a physician information letter to convey to their primary healthcare providers if they decided to get vaccinated.

Stages 3: Three weeks after this briefing, mothers were asked six questions related to the second part of the survey via phone contact. Mothers who had not yet discharged their babies or were undecided about vaccination were contacted again 3 weeks later. Additionally, when the newborns turned 3 months old, communication was re-established with mothers to record the vaccination status of the babies at 1 and 2 months. In Türkiye, within the scope of the expanded immunization program, Hepatitis B vaccine is given to babies at birth and at the 1st and 6th months of life; in the 2nd month, conjugated pneumococcal vaccine, tuberculosis, and DTaP-IPV-Hib vaccines are administered. Information about the mothers’ vaccinations during pregnancy was obtained from the electronic record system.

### 2.3. Survey and Information Form

The survey form and family information form to be used for data collection were prepared based on scientific studies in the field [2,11,12,13]. These forms were evaluated and received recommendations from physicians experienced in four different specialties (social pediatrics, pediatric infectious diseases, pediatric allergy immunology, and neonatology).

The survey form (given as the Supplementary Materials of this article) consisted of two parts: the first part, with 31 questions, was administered face-to-face, while the second part, with 6 questions, was administered over the phone. In total, there were 37 questions.

In the first part, there were 13 questions related to sociodemographic characteristics, 5 questions about pregnancy visits and vaccinations, 6 questions about mothers’ knowledge levels regarding pertussis disease, 6 questions about knowledge levels regarding pertussis vaccines, and 1 question about vaccination intentions. For the questions aimed at determining knowledge levels, response options were provided as Yes–No–Don’t know. Scoring was conducted in a way that correct answers received 1 point, while “Don’t know” and incorrect answers received 0 points. The sum of the scores for the six questions about pertussis disease was named Disease Knowledge Level (DKL) and the sum of the scores for the six questions about pertussis vaccines was named Vaccine Knowledge Level (VKL).

The second part aimed to determine whether verbal and written information was sufficient, the status of the mother receiving the Tdap vaccine, where the vaccine was administered, the situation of another family member receiving the Tdap vaccine, and the reasons for choosing to vaccinate or not.

Birth weight, the gestational age of newborns, and the duration of hospitalization were recorded from medical records. In cases where there were twins, only the information for the first-born (numbered as 1) was included in the evaluation, resulting in a total of 232 newborns, including 12 sets of twins. Demographic characteristics were considered based on data from only one newborn. However, vaccination status was provided for all newborns (n = 232).

### 2.4. Pilot Study

A pilot study was conducted with 10 mothers to assess the comprehensibility of the survey questions and the text in the information form. As a result of the pilot study, for the question “Which vaccines recommendations did you receive for during your pregnancy?” in the survey form, answer options “I don’t remember” and “I didn’t receive any recommendations” were added. The question “Which vaccines did you get during your pregnancy?” now included the response option “I did not get any vaccines at all”. Additionally, in the family information form, the section regarding breastfeeding mothers’ ability to get vaccinated was reorganized and emphasized, highlighting it in bold.

### 2.5. Sample Size

The literature review did not reveal a similar study from which we could obtain sample analysis. However, a preliminary calculation was made based on the Cohen’s d effect size before this study. Calculations were performed considering Cohen’s d = 0.50 and Cohen’s d = 0.20, resulting in sample sizes of 51 and 310, respectively. Given the belief that any number within this range would be sufficient, this research aimed to reach a sample of approximately 220 mothers, with a 5% margin of error, 80% power, and the anticipated effect size.

### 2.6. Statistical Analysis

Statistical analysis was performed using SPSS software version 21 (SPSS Inc., IBM, Armonk, NY, USA). Categorical variables were presented with frequencies, while continuous numerical variables were presented with mean values along with their standard deviations. Normal distribution was assessed before data analysis. Two methods were used for evaluating normal distribution. The first method involved assessing the kurtosis and skewness values and the second method considered the sample size. If the sample size was less than 50, the Shapiro–Wilk test was used; if the sample size was 50 or higher, the Kolmogorov–Smirnov test was employed to evaluate normal distribution. The data were considered to have a normal distribution if the skewness and kurtosis values were between +2 and −2, or if the *p*-value from both the Shapiro–Wilk and Kolmogorov–Smirnov tests was greater than 0.05. However, as our data did not meet both conditions, the non-parametric Mann–Whitney U test was used. The chi-square test was employed for comparisons between categorical variables, with *p*-values less than 0.05 (*p* < 0.05) considered statistically significant. The dependent variables were receiving the Tdap vaccine and vaccine intention. Since only five mothers received the Tdap vaccine, the relationship between receiving the vaccine and descriptive variables could not be analyzed. Analyses between vaccination intention and descriptive variables were performed using the chi-square test.

## 3. Results

### 3.1. Sociodemographic Characteristics of the Mothers and Newborns

The average age of mothers was 28 ± 5.6 years, with 35% having graduated from high school. Approximately 73% of the mothers were homemakers and 75.5% did not work in any job prior to the birth. The average age of fathers was 32 ± 6.2 years, with 34.5% having completed high school. In addition, 62% of the fathers worked as laborers.

When evaluating total monthly income of the family, it was observed that 20.5% of families had an income equal to or below the minimum wage. When classified according to their places of residence, 55% lived in the city center, 34% in districts, and 11% in villages. Detailed information on sociodemographic characteristics is presented in Table 1.

Newborns (n = 220) had birth weights ranging from 600 to 4800 g, with an average of 2625 ± 855 g (median = 2745 g). Their gestational ages ranged from 24 to 41 weeks, with an average of 36.2 ± 3.5 weeks (median = 37 ± 3). When evaluated based on the weeks of gestation, 47% of the babies were born before 37 weeks, categorizing them as preterm.

### 3.2. Pregnancy Follow-Ups and Vaccination Status

During the pregnancy period, 96% of mothers attended their prenatal care regularly. Regarding prenatal care, 88% mentioned that they received vaccinations, and 81% (n = 179) specifically had tetanus vaccinations. According to the vaccination record system checks, 82% (n = 180) were vaccinated with Td. Throughout the pregnancy follow-ups, none of the mothers received a recommendation for Tdap vaccination, and none were vaccinated with Tdap. Vaccination recommendation for mothers during pregnancy was mostly provided by midwives/nurses at the family health center (60%) and family physicians (32%) (Table 2). It was found that 22% of the mothers were recommended to have the COVID vaccine during pregnancy, and 26% received the COVID vaccine during pregnancy. The data regarding mothers’ pregnancy follow-ups and vaccinations are presented in Table 3.

### 3.3. Mothers’ Knowledge of Pertussis Disease and Vaccinations

Pertussis disease knowledge levels, as measured by the questions asked, are shown in Table 4. While 90% of mothers were unaware that adults could also contract pertussis, 88% did not know how the disease was transmitted to infants. Approximately 68.2% of mothers could not provide correct answers to any of the questions, resulting in a Disease Knowledge Level (DKL) score of 0. For all vaccine-related questions, the rates of mothers providing correct answers ranged from 5% to 23% (Table 4). Approximately 68% of mothers had a Vaccine Knowledge Level (VKL) score of 0.

The questions assessing knowledge were asked before verbal and written information provided by the researcher. Therefore, they reflect the baseline knowledge levels of mothers about pertussis. The adequacy of the information provided by the researcher and written information forms given to mothers was evaluated. Of the 220 mothers, 208 (95%) stated that information received from the doctor was sufficient and 206 (94%) indicated that information in the family information form was adequate.

### 3.4. Vaccination Intention

When asked about their intentions regarding vaccination, 60% of mothers expressed indecision, 36% considered getting vaccinated, and 5% stated that they did not consider vaccination. To assess the factors influencing vaccination intentions, a comparison was made between those considering vaccination and those who were undecided. Since there were a limited number of individuals who did not consider vaccination, they were grouped with the undecided. When comparing those considering vaccination and those not considering vaccination, no significant differences were found in terms of parental age, parental disease and vaccine knowledge levels, baby’s birth weight and weeks of gestation, duration of hospitalization, and the presence of siblings attending school (*p* > 0.05). Similarly, there were no significant differences between those considering vaccination and others in terms of parental education level, parental occupation, maternal employment status, place of residence, and regular attendance at prenatal check-ups (*p* > 0.05). When comparing the vaccination intentions of mothers of babies born at less than 32 weeks and those born between 32 and 37 weeks, no significant difference was found between the two groups (*p* > 0.05).

### 3.5. Reasons for Getting Vaccinated

Only 2% (n = 5) of the participating mothers received the Tdap vaccine. Mothers who received Tdap were able to obtain it from the pharmacy and then get vaccinated at the Family Health Center. No one else in the family, besides themselves, had received the Tdap vaccine. The details of the mothers who received Tdap and their babies are provided in Table 5.

Two individuals mentioned that they received the vaccine because pertussis can progress severely in babies, while the other two did so because they believed pertussis could be transmitted from the mother to the baby, and one person chose to be vaccinated based on information received from a doctor.

### 3.6. Reasons for Not Getting Vaccinated

The reasons for mothers not getting vaccinated are listed in Table 6. The most common reasons among the factors for not getting vaccinated were not finding time (24.2%) (due to health issues of the baby and herself, doctor appointments, etc.) and vaccine cost (22.8%). Among 22 individuals grouped under “Other Reasons”, 13 mentioned not finding the vaccine at the pharmacy, 6 stated that there was no one around them who had received this vaccine, 1 mentioned the pharmacy being too far away, and 2 individuals reported not getting vaccinated because this vaccine was not included in Ministry of Health’s routine vaccination schedule.

When newborns were grouped as preterm and term based on their weeks of gestation, a significant difference was observed in terms of prematurity between those who did not get vaccinated due to a lack of time and other reasons (*p* = 0.012). Of the mothers who expressed not being able to get vaccinated due to a lack of time, 63% had premature newborns.

When compared in terms of monthly family income, a significant difference was found between those who cited vaccine cost as the reason for not getting vaccinated and those who did not get vaccinated for other reasons (*p* = 0.008) (Table 7). In interim report assessments conducted every 6 months during this research, it was determined that there was an increase of 11%, 22%, and 28% among those who did not get vaccinated due to vaccine cost.

Among the mothers in this study, 14 were healthcare workers, and none of them had received the Tdap vaccine. When the reasons for healthcare workers not getting vaccinated were compared with the reasons in the general group, the percentage of those not getting vaccinated due to perceiving their babies not being at risk for pertussis was 13% in the general group, while among healthcare worker mothers, this rate increased to 21%.

Of the 78 individuals with a positive intention to get vaccinated, 75 had not received the vaccine. When the reasons in this group were examined, the primary reason was the cost of the vaccine (28%). Other reasons are summarized in Table 8.

### 3.7. Characteristics of the Vaccination Status of Newborns and Their Siblings

When the vaccination status of newborns was assessed at 3 months old for the 1st and 2nd months (n = 232), 229 infants (99%) were fully vaccinated according to their age. Two infants had not been vaccinated due to their family’s vaccine hesitancy, and one infant’s vaccination schedule had been temporarily interrupted due to health issues after receiving the 1st-month vaccine.

The vaccination status of siblings (n = 229) was evaluated based on maternal statements. It was reported that all siblings, except for one child from a vaccine-hesitant family, were appropriately vaccinated for their age.

## 4. Discussion

This study, to our knowledge, is the first research on the implementation of the cocoon strategy for pertussis in the neonatal intensive care unit in Türkiye. Due to the ongoing infant deaths related to pertussis in our country, we designed this study to understand the challenges faced in the healthcare system to improve vaccination rates in this area. The vaccination rates with Tdap within the cocoon strategy for mothers after childbirth (n = 5, 2%) were found to be very low. When the reasons for non-compliance with the cocoon strategy were examined, it was observed that mothers faced issues such as not finding time to get vaccinated, vaccine costs, and accessibility to the vaccine. On the other hand, it was found that vaccination rates with Td during pregnancy in our country are in a good state, and the inclusion of the Tdap vaccine among routine pregnancy vaccines suggests that overcoming barriers in cocoon implementation will increase vaccine coverage.

Sociodemographic characteristics are known to influence vaccine acceptance [14]. Studies examining the feasibility of Tdap within the cocoon strategy have found that the parents’ education level, age, occupation, and their number of children can affect vaccine acceptance [15,16]. While some studies have reported higher vaccine acceptance among mothers with a higher education level [11], others have indicated higher vaccine acceptance among individuals with a lower education level [15]. In our study, there was no significant difference in sociodemographic characteristics such as parental age, education level, occupation, and the number of other children in the family when comparing those who intended to get vaccinated and others. The variability in sociodemographic results may be attributed to differences in study designs, the presence of pertussis epidemics in countries during the study period, and variations in local health authorities’ Tdap vaccination practices.

In our country, the Public Health Directorate has prepared a Prenatal Care Guide for all pregnant women. At the first antenatal visit, it is recommended to provide information to mothers about Td, hepatitis B, and influenza vaccines. However, there is no information about Tdap in the guide [17]. Among a group of participants at a pregnancy school, where mothers can voluntarily participate, information about pertussis and Tdap vaccines was provided by a pediatrician during sessions, and in follow-ups, it was observed that none of the pregnant women received the Tdap vaccine [18]. However, a study conducted in Taiwan showed an increase in Tdap vaccine acceptance with the increase in prenatal follow-up. In this study, healthcare professionals such as nurses, doctors, and pharmacists who came into contact with pregnant women were trained on the Tdap vaccine. Pregnant women were informed about Tdap by a nurse during their third trimester visits, and educational videos on the subject were shared with them [19]. In our study, 96% of the mothers stated that they attended prenatal visits regularly (four times or more). However, regular follow-up did not affect vaccine intention. It is considered that the absence of maternal vaccination and the cocoon strategy in the educational content during prenatal follow-up may contribute to this situation.

Increased knowledge and awareness about diseases is thought to positively influence the intention to get vaccinated [20]. In a study among pregnant women, only 5% of the mothers reported having heard about pertussis disease before [21]. A study among healthcare workers showed that the knowledge level about pertussis vaccines was low, but there was a significant relationship between increased knowledge about the vaccine and positive vaccine intention [22]. In a survey conducted among pregnant women in Mexico, it was found that 16% had heard about pertussis disease before, and hearing about pertussis disease previously positively influenced vaccine intention [20].

In Australia, 93% of mothers had heard about pertussis disease before, 95% knew that pertussis could cause severe illness in infants, and 71% knew that adults could also contract pertussis. Regarding vaccination status, 70% of mothers were reported to be vaccinated with Tdap [16]. In our study, the knowledge rates among mothers about the severity of pertussis in infants (12%), the possibility of adults contracting pertussis (9%), and the usual transmission of pertussis to infants from their close contacts (7%) were found to be quite low compared to the literature. The rates of mothers knowing that the vaccine could be administered during pregnancy or breastfeeding (5%) and that vaccinating the mother could protect the baby (10%) were also low. These deficiencies in knowledge were considered to have an effect on the very low vaccination rates among mothers (2%).

France is a country where the cocoon strategy has been recommended by national health authorities since 2004. A decade after its implementation, mothers’ pertussis vaccination rates increased from 21.9% to 61% between 2009 and 2014. In France, healthcare professionals from whom vaccine recommendations were received were, respectively, general practitioner (56.5%), obstetrician–gynecologist (46.1%), midwife (41.6%), and pediatrician (19.2%) [23]. In a study by Wong et al., information about influenza and pertussis vaccines recommended during pregnancy was primarily obtained from healthcare professionals [11]. In our study, it was observed that midwives or nurses in Primary Health Care Centers (PHCC) were the primary source of information (60%) for vaccinations during pregnancy, followed by family physicians (32%) and obstetrician–gynecologists (20%). It is known that the attitudes of healthcare workers also play a role in getting vaccinated. There have been instances where individuals, despite being recommended Tdap vaccination by healthcare professionals, did not get vaccinated because their own doctor did not support the vaccination [18,24]. Tdap was not among the vaccines recommended to mothers during pregnancy. On the other hand, there were mothers who did not get vaccinated with Tdap because their family physician or midwife/nurse at the PHCC stated that the vaccine was unnecessary or not in use (n = 7). These findings suggest the crucial role of midwives, nurses, and family physicians working in primary care in improving pregnancy vaccinations and the cocoon strategy in our country.

A known factor that influences vaccine uptake is the cost and accessibility of vaccine administration [25]. In programs where vaccines are free, and vaccine services are easily accessible, vaccination rates have been found to be quite high [26,27,28]. In a study conducted in California, the researchers utilized a room in postpartum services as the “Family Tdap Vaccination Room” and provided free Tdap vaccinations to visitors (siblings, grandparents, etc.) in the vaccination room. This practice increased the vaccination rate of the entire family who would come into contact with the baby from 29% to 76% [28]. In our study, mothers had to pay for the vaccine when they wanted to get vaccinated, and the cost was the second-highest factor (22.8%) among the reasons for not getting vaccinated. Among those who had a positive intention to get vaccinated but did not get vaccinated, the cost was the primary reason (28%). It is considered that economic conditions significantly influence vaccine practices, and providing free access to vaccines could lead to a substantial increase in vaccination rates.

We believe that understanding the reasons for individuals with a positive intention toward vaccination but not getting vaccinated would provide more guidance for implementation. Among those who intended to get vaccinated but did not, the top three reasons were found to be vaccine cost, lack of time, and an inability to obtain the vaccine from the pharmacy, respectively. In another study, the rate of those unable to get vaccinated due to a lack of access to the vaccine was 2.6% [11], while in our study, it was 17%. Lack of vaccine access was not mentioned as a reason in other studies [16,19,29]. These data suggest that ensuring free accessibility to the vaccine in the planning of the cocoon strategy in our country could contribute to increased vaccination rates.

In a study conducted on childhood vaccinations in our country, although 30% of parents did not know the names of any vaccines from the Ministry of Health’s routine vaccination schedule, the majority of infants (94%) were vaccinated. Excluding those whose vaccines were postponed due to illness, only 1.4% of babies were found not to be vaccinated without any reason. The number of those who received non-routine vaccinations was determined to be 15.6% [30]. In our study, 99% of babies were fully vaccinated according to their age. It was observed that the routine childhood vaccination schedule has been successfully implemented in our country. Non-routine childhood vaccinations are also more widely accepted compared to the cocoon strategy. This difference may be attributed to non-routine vaccines having obtained approval in our country earlier than the Tdap vaccine and pediatricians more widely recommending these vaccines

This research was conducted during the COVID-19 pandemic. In our country, access to influenza vaccines during the pandemic was limited to individuals with chronic illnesses and those aged 65 and over. Therefore, the evaluation did not include the influenza vaccination, which is another vaccine recommended within the cocoon strategy.

## 5. Conclusions

In terms of implementation of the cocoon strategy, the foremost reasons include vaccine cost/accessibility and mothers not having time to get vaccinated. It was observed that mothers attended regular pregnancy follow-up (96%) and were largely vaccinated with Td (82%). Newborns and siblings in this study were observed to be 99% fully vaccinated according to their age and there was good compliance with the expanded immunization program. Considering the high vaccine acceptance rates in pregnancy and childhood vaccinations, we believe that addressing the identified issues of cost and accessibility by including the vaccine in the national vaccination schedule could enhance the feasibility of implementing Tdap vaccination for pregnant women.

## Figures and Tables

**Table 1:** Sociodemographic characteristics of the parents.

Characteristics	n = 220	%
Education of Mother		
Primary school	32	14.5
Middle school	67	30.5
High school	77	35
University	44	20
Occupation of Mother		
Teacher	9	4.1
Healthcare worker	14	6.4
Laborer	23	10.5
Housemaker	161	73.2
Public officer	3	1.4
Other	10	4.5
Employment status of mother		
Yes	54	24.5
No	166	75.5
Education of Father		
Primary school	40	18.2
Middle school	65	29.5
High school	76	34.5
University degree	37	16.8
Postgraduate	2	0.9
Occupation of Father		
Teacher	7	3.2
Healthcare worker	2	0.9
Public officer	18	8.2
Laborer	137	62.3
Shop owner	31	14.1
Other	25	11.4
Residence		
City center	122	55.5
Town	74	33.6
Village	24	10.9
Family income *		
Minimum wage or below	45	20.5
1 to ≤3 fold	112	50.9
3 to ≤5 fold	24	10.9
5 fold or above	7	3.2
Not specified	32	14.5

* Compared to the minimum wage.

**Table 2:** Healthcare professionals from whom mothers receive vaccination recommendations during pregnancy.

Healthcare Professionals *	n	%
Midwife and nurse	131	59.5
Family physician	71	32.2
Obstetrics and gynecology	44	20
Not informed	12	5.4
All of them	2	0.9

* There were those who were informed by multiple individuals.

**Table 3:** Mothers’ pregnancy follow-up and vaccination status.

Pregnancy Follow-Up and Vaccination Status	n = 220	%
Regular prenatal visit (four times or more)		
Yes	211	95.9
No	9	4.1
Recommended vaccination during pregnancy		
Td	116	52.7
COVID-19	49	22.2
Don’t remember	65	29.5
Didn’t receive any recommendation	30	13.6
Hepatitis B	3	0.9
Influenza	1	0.5
Vaccinations administered during pregnancy *		
Td	179	81.3
COVID-19	41	18.6
Not vaccinated	27	12.2
Don’t remember	7	3.1
Hepatitis B	3	1.3
Influenza	1	0.5
Vaccinations administered during pregnancy **		
Td		
Vaccinated	166	75.5
Vaccinated during previous pregnancy	14	6.4
Not vaccinated	12	5.5
Unreachable	28	12.7
COVID-19		
Vaccinated before pregnancy	22	10
Vaccinated during pregnancy	58	26.4
Vaccinated after giving birth	22	10
Not vaccinated	90	40.9
Unreachable	28	12.7

* According to mothers’ statement; ** According to vaccination record system.

**Table 4:** Mothers’ level of knowledge about pertussis disease and vaccinations (n = 220).

	Yes n (%)	No n (%)	Don’t Know n (%)
Pertussis Disease Knowledge Level			
Pertussis is transmitted from person to person through droplets produced during coughing and sneezing.	45 (20)	8 (4)	167 (76)
Pertussis is more severe in premature babies, especially in infants under 6 months of age.	27 (12)	1 (1)	192 (87)
Pertussis can affect the lungs, heart, and brain.	35 (16)	1 (1)	184 (84)
Pertussis can cause serious consequences, ranging from hospitalization to death.	38 (17)	3 (2)	179 (81)
Pertussis is transmitted to newborn babies, usually from their parents or siblings who have whooping cough.	16 (7)	10 (5)	194 (88)
Adults can get whooping cough, which is accompanied by a long-term cough.	20 (9)	2 (1)	198 (90)
Pertussis Vaccine Knowledge Level			
Pertussis vaccine is very effective in preventing whooping cough.	50 (23)	4 (2)	166 (75)
Pertussis vaccine is included in the routine childhood vaccination schedule implemented by the Ministry of Health.	28 (13)	10 (4)	182 (83)
Tdap vaccine can be given to pregnant women.	10 (5)	7 (3)	203 (92)
Tdap vaccine can be given to breastfeeding mothers.	10 (5)	5 (2)	205 (93)
Vaccination of the mother with pertussis vaccine after birth is effective in protecting the baby from this disease.	21 (10)	7 (3)	192 (87)
Benefits of the vaccine far outweigh its possible side effects.	35 (16)	5 (2)	180 (82)

**Table 5:** Characteristics of mothers and infants who received the Tdap vaccine.

	Case 1	Case 2	Case 3	Case 4	Case 5
Age	29	28	33	29	32
Education	University	Middle school	Middle school	High school	Middle school
Employment status	No	No	No	Yes	No
Occupation	Housemaker	Housemaker	Housemaker	Public officer	Housemaker
DKL *	0/6	0/6	1/6	1/6	3/6
VKL **	1/6	0/6	2/6	4/6	1/6
Vaccine intention	Indecision	Positive	Positive	Indecision	Positive
Birth weight	3800 g	2050 g	2350 g	3820 g	1800 g
Gestational week	39	33	38	39	38
Duration of stay in hospital	10 days	15 days	7 days	3 days	10 days

* Disease Knowledge Level; ** Vaccine Knowledge Level.

**Table 6:** Reasons for mothers not getting the Tdap vaccine.

	N	%
Lack of time	52	24.2
Vaccine cost	49	22.8
Thought there may be side effects related to vaccine	24	11.2
Thought vaccine will be ineffective	3	1,4
Told her not to get vaccinated		
Spouse	15	6.9
Family physician	5	2.3
Nurse/midwife	2	1
Thought contraindications related to breastfeeding	4	1.9
Fear of injection	3	1.4
Babies not being at risk for pertussis disease	28	13.0
Not wanting to go to a healthcare institution due to pandemic	8	3.7
Others *	22	10.2
Total	215	100.0

* Mentioned not finding the vaccine at the pharmacy (13); Stated that there was no one around them who had received this vaccine (6); Mentioned the pharmacy being too far away (1); Vaccine was not included in Ministry of Health’s routine vaccination schedule (2).

**Table 7:** Economic status of those who did not get vaccinated due to the cost of vaccination and other reasons.

	Other Reason n (%)	Vaccine Cost n (%)	Total n (%)
Family Income			
Minimum wage or below	27 (16.3)	18 (36.7)	45 (20.9)
1 to ≤3 fold	88 (53)	23 (46.9)	111 (51.6)
3 to ≤5 fold	21 (12.7)	1 (2)	22 (10.2)
5 fold or above	6 (3.6)	0 (0)	6 (2.8)
Not specified	24 (14.5)	7 (14.3)	31 (14.4)
Total	166 (100)	49 (100)	215 (100)

Chi-square test, *p* = 0.008.

**Table 8:** Reasons for not vaccinating among those who have a positive intention toward vaccination.

Positive Vaccination Intention and Reasons for Not Vaccinating	n	%
Vaccine cost	21	28
Lack of time	17	22.7
Thought there may be side effects related to vaccine	3	4.0
Thought the vaccine will be ineffective	1	1.3
Told her not to get vaccinated (spouse, nurse/midwife/family physician)	11	14.7
Fear of injection	1	1.3
Babies not being at risk for pertussis disease	7	9.3
Not wanting to go to a healthcare institution due to pandemic	1	1.3
Not finding the vaccine at the pharmacy	13	17.3
Total	75	100.0

## Data Availability

The datasets used and/or analyzed during the current study are available from the corresponding author upon reasonable request.

## Supplementary Materials

The following supporting information can be downloaded at: https://www.mdpi.com/article/10.3390/vaccines12030319/s1, Survey Form.
