# Supplementary material for: Factors Affecting the Implementation and Acceptance of the Cocoon Strategy in the NICU in a Tertiary Center in Türkiye
Source: Vaccines (Basel). 2024 Mar 18;12(3):319. doi: 10.3390/vaccines12030319 (PMC10976068; doi:10.3390/vaccines12030319)
Supplement: Supplementary file 1 [file vaccines-12-00319-s001.zip › vaccines-2902155-supplementary.pdf]

Survey Form

Do you have any conditions that might prevent you from getting vaccinated?

1. (…) Serious allergic reaction to any vaccine, drug, or substance
2. (…) Coma, confusion, or prolonged seizures occurring within 7 days after previous DTaP vaccination
3. (…) Guillain-Barre syndrome (neurological disease that developed within the 6 weeks after previous vaccinations)
4. (…) Epilepsy or other neurological disorders

Baby's Birth Weight:……………g Baby's Gestational Age:…………… Length of Stay:……. days Baby's Diagnosis: Prematurity and its complications (…) Hyperbilirubinemia(…) Dehydration (…) Sepsis(…) Respiratory distress(…)

Mother's full Name: ……………………………. Phone number: ……………………
Date: …/…/2021

Part 1:

1. Age: …..
2. City of residence: ………………….
3. Place of residence: City center (…) Town (…) Village (…)
4. Education level:

   Primary school (...) Middle school (...) High school (...) University (...) Postgraduate (…)
5. Occupation:

   Teacher (...) Healthcare Worker (…) Laborer (…) Public officer (...) Housemaker (...), Other: ………………………….
6. Employment status before pregnancy:

   Employed (...) Unemployed (…)
7. Age of your spouse: …..
8. Education level of your spouse:

   Primary school (...) Middle school (...) High school (...) University (...) Postgraduate (…)
9. Occupation of your spouse:

   Teacher (...), Healthcare Worker (…), Public officer(…), Laborer (...), Shop owner(...), Other: ………………………….

10. Total monthly income of your family:

2324 TL and below (…) 2325 TL - 6899 TL (…) 6900 TL - 11599 TL (…) 11600 TL and above (…)

11. How many children do you have apart from the baby currently hospitalized in the neonatal intensive care unit? ………

12. Do you have children attending school?

Yes (…) No (…)

13. Are your other children fully vaccinated according to the Ministry of Health Vaccination Schedule?

Yes(...) No/Incomplete vaccinations(…)

Vaccination in pregnancy:

14. Did you have regular follow-ups during this pregnancy? (If you visited a doctor 4 times or more during pregnancy, please mark Yes)

Yes (…) No (…)

15. Did you receive any vaccinations during this pregnancy?

Yes (…) No (…) I don't remember (...)

16. What vaccinations were recommended to you during this pregnancy? ………………………………………. I don't remember (...) I did not receive any recommendations(...)

17. Which vaccinations did you receive during this pregnancy?

Tetanus diphtheria (...) Flu (…) Other(…………...) I did not receive any vaccinations (….)

18. Who informed you about the vaccinations in pregnancy?

Obstetrician-gynecologist (….)
Family physician (….)
Nurse or midwife at the family health center (….)
Other (…………………………………………)
I was not informed (….)

Knowledge level about pertussis:

19. Pertussis disease is transmitted through droplets which are generated by coughing and sneezing.

Yes (...) No (...) I don't know (…)

20. Pertussis disease tends to be more severe in infants under 6 months, especially in premature (born before term) babies.

    Yes (…) No (...) I don't know (…)

21. Pertussis disease can affect the lungs, heart, and brain.

    Yes (...) No (...) I don't know (…)

22. Pertussis disease can lead to serious consequences ranging from hospitalization to death. Yes (...) No (...) I don't know (…)

23. Pertussis disease is transmitted to newborn babies most commonly from parents or siblings with pertussis.

    Yes (...) No (...) I don't know (…)

24. Adults can experience pertussis disease characterized by prolonged coughing.

    Yes (...) No (...) I don't know (…)

Knowledge Level about Pertussis Vaccine:

25. The pertussis vaccine is highly effective in preventing pertussis disease.
    Yes (...) No (...) I don't know (…)

26. The Ministry of Health's vaccination schedule includes the pertussis vaccine for infants.
    Yes (...) No (...) I don't know (…)

27. There is no contraindication for administering the pertussis vaccine to pregnant women.
    Yes (...) No (...) I don't know (…)

28. There is no contraindication for administering the pertussis vaccine to breastfeeding mothers.
    Yes (...) No (...) I don't know (…)

29. Immunizing the mother with the pertussis vaccine after childbirth is effective in protecting the baby from this disease.
    Yes (...) No (...) I don't know (…)

30. The benefits of the vaccine outweigh the possible side effects.
    Yes (...) No (...) I don't know (…)

Vaccination intention

31. **After receiving the information, the intention about getting the pertussis vaccine:**
    Intends to (…)   Doesn't intend to (…)   Currently undecisive(…)

Part 2:

3 WEEKS AFTER THE INFORMATION SESSION AND FIRST SURVEY, IN THE FOLLOW-UP PHONE CALL:

1. Did you find the information provided by your doctor sufficient?

   Yes (…)   No(…)

2. Did you find the Family Information Form provided to you sufficient?

   Yes (…)   No(…)

3. Did you receive the Tdap vaccine?

   Yes (…)   No(…)   I haven't decided yet(…)

4. Where did you get vaccinated? ………………………………………………

5. Did you get anyone else in your family vaccinated besides yourself?

   Sibling (…)   Father (…)   Grandmother (…)   Grandfather (…)   Other:…….……..

6. If your answer to question 3 is Yes, what is the reason for your vaccination?

   1) I know that pertussis can be severe in babies.
   2) Because transmission of pertussis to babies is usually from mothers or close contacts at home.
   3) Because my doctor recommended it.
   4) Based on the recommendation of other mothers who were vaccinated.
   5) Other: ………………….

   If your answer to question 3 is No, what is the reason for not getting vaccinated?

   1) I didn't have time.
   2) I didn't want to go to healthcare facilities due to the pandemic.
   3) I think the vaccine may have side effects.
   4) I think the vaccine will not be effective.
   5) My family doctor/pharmacist/other (…………) advised me not to get vaccinated.
   6) Because I'm breastfeeding, it might have side effects on my baby.
   7) Because I'm afraid of injections.
   8) I don't think my baby is at risk for pertussis.
   9) I couldn't afford the cost of the vaccine.
   10) Other: ……………………
